# Ulcerative colitis (UC) in the elderly: diagnostic and therapeutic considerations

**DOI:** 10.3389/fragi.2026.1815043

**Published:** 2026-08-04

**Authors:** Łucja Wróbel, Ewa Małecka-Wojciesko

**Affiliations:** Department of Digestive Tract Diseases, Medical University of Lodz, Lodz, Poland

**Keywords:** elderly, immunosenescence, inflammatory bowel disease, polypharmacy, ulcerative colitis

## Abstract

Ulcerative colitis (UC) demonstrates a bimodal incidence distribution, with a significant second peak and the highest prevalence observed in individuals aged ≥60 years. The pathogenesis of elderly UC involves immunosenescence—manifested by Th17/Treg imbalance and the accumulation of cells with a pro-inflammatory senescence-associated secretory phenotype (SASP)—alongside age-related dysbiosis, increased intestinal permeability, and mitochondrial dysfunction that impairs mucosal regeneration. Clinically, older patients more frequently present with left-sided colitis, characterized by anemia and weight loss, while abdominal pain is less common. Managing geriatric UC is challenging due to inadequate colonoscopy preparation and complex differential diagnosis (e.g., colorectal cancer, ischemic colitis). Furthermore, older adults face a fivefold increased risk of *Clostridioides difficile* infection and a sharply rising risk of colorectal cancer if diagnosed after 70 years of age. While 5-aminosalicylic acid remains the first-line therapy, it requires renal monitoring. Conversely, steroids increase infection and osteoporosis risks, thiopurines are restricted due to myelotoxicity and malignancy risks, and among biologics, vedolizumab offers better safety than anti-TNF agents. Ultimately, managing UC in older adults requires a comprehensive, multidisciplinary approach that accounts for multimorbidity, polypharmacy, nutritional status, and mental health to optimize pharmacotherapy and mitigate high-risk surgical interventions.

## 1 Introduction

Ulcerative colitis (UC) is part of the spectrum of inflammatory bowel disease (IBD). In patients aged 60 years and older, UC represents a significant healthcare challenge due to age-related physiological changes and the high burden of comorbidities, which may affect both disease course and therapeutic decision-making. At the same time, advances in understanding of UC pathophysiology have led to the development of increasingly effective, targeted therapeutic options, underscoring the importance of optimized management in older patients. To ensure methodological clarity throughout this review, the study population is strictly defined based on chronological age and disease duration. First, “older adults” or “elderly patients” are defined using the widely accepted international threshold of ≥60 years of age. Second, a crucial clinical distinction is maintained between two separate patient populations: (1) elderly-onset UC (E-UC), defined as patients whose initial diagnosis occurred at or after the age of 60, and (2) adult-onset UC aging with long-standing disease (A-UC), defined as patients diagnosed before the age of 60 who have subsequently transitioned into the geriatric age group. Consequently, to provide an accurate clinical picture, the epidemiology, colorectal cancer risk, therapeutic safety profiles, and postoperative surgical outcomes are discussed separately for these two distinct groups throughout the relevant sections of this manuscript.

### 1.1 Epidemiology

Over the years, the prevalence of UC has continued to rise. In Poland, between 2009 and 2020, among patients aged 60–69 years, the highest UC prevalence was observed; the next highest was among patients aged 20–29 years (Zagórowicz et al., 2022). While large cohort studies suggest that overall life expectancy in UC patients approaches that of the general population (Höie et al., 2007), this trajectory is highly nuanced in older adults. In the geriatric population, particularly within the first few years following a late-onset diagnosis, survival rates can be compromised by aggressive disease courses, a higher susceptibility to serious infections, and increased perioperative mortality. UC demonstrates a bimodal incidence distribution, with a single peak in the 20–29 age group and a second peak in individuals ≥60 years (Rubi et al., 2019; da Silva et al., 2014). Therefore, UC prevalence in this older age group reflects a combination of late-onset cases and patients with long-standing disease.

### 1.2 Pathophysiology in older adults

#### 1.2.1 Age-related immune changes (immunosenescence)

Immunosenescence, a process of immunological remodeling, is considered to be involved in the pathomechanism of elderly-onset inflammatory bowel disease (IBD). With thymic involution, there is an increased accumulation of memory T cells and a reduction in naive T cells, resulting in diminished responsiveness to newly encountered antigens. Additionally, the ratio of T helper 17 cells to regulatory T cells, as well as the CD4/CD8 ratio, increases. An imbalance between Th1 and Th2 subsets, as well as between regulatory and effector T cells, is also observed (Zhang et al., 2025a). T lymphocytes with a pro-inflammatory profile accumulate in peripheral blood and tissues, exhibiting a senescence-associated secretory phenotype (SASP) and secreting cytokines such as IL-6, IL-1β, and TNF-α, contributing to a chronic low-grade inflammatory environment (“inflammaging”) (Franceschi et al., 2018; Eder et al., 2023). Immunosenescence in B lymphocytes includes a decline in lymphopoiesis and reduced diversity, leading to diminished avidity of IgG and IgA antibodies, particularly those involved in mucosal immunity (Frasca et al., 2020). Senescent B cell populations, including memory and age-associated B cells, also adopt a SASP, secreting pro-inflammatory cytokines and autoantibodies, such as anti-neutrophil cytoplasmic antibodies (ANCA), which may contribute to mucosal tissue damage in UC (Frasca et al., 2020; Yue et al., 2022). Aging is accompanied by dendritic cell dysfunction in the intestinal lamina propria and secondary lymphoid tissues, with reduced phagocytosis of apoptotic cells and increased basal secretion of pro-inflammatory cytokines, thereby promoting local autoimmune reactions and chronic inflammation (Chougnet et al., 2015).

#### 1.2.2 Age-related microbiome alterations

Age-associated changes in gut microbiota composition and dysbiosis have been heavily implicated in the pathogenesis of IBD. Under physiological conditions, the intestinal microbiota remains in homeostasis with the immune system, with bacteria prevented from penetrating colonocytes by a thick mucus layer and an intact intestinal barrier. With aging, the bacterial diversity declines along with a reduction in the abundance of beneficial commensal taxa (such as *Bacteroidetes, Firmicutes, and Actinobacteria*). These pyla produce key metabolites, including short-chain fatty acids (SCFAs), like butyrate, which attenuates intestinal inflammation by promoting the expansion of regulatory T cells (Tregs) and modulating the function of regulatory B cells (Bregs). In age-related dysbiosis, pathobionts or facultative anaerobes, such as *Salmonella enterica* and *Escherichia coli*, may expand dysbiotic conditions. Furthermore, those taxa can release enterotoxins that induce oxidative stress in epithelial cells and damage colonocytes, thereby compromising mucosal function. Increased intestinal permeability associated with age-related dysbiosis enable the translocation of PAMPs (Pathogen-Associated Molecular Patterns) into the systemic circulation, triggering an immune response and persistent immune response (Meng et al., 2023; Xie et al., 2025; Qiu et al., 2022). Dysbiosis in the elderly is also driven by reduced dietary fiber intake and polypharmacy. A nationwide Polish study showed that in 2019, polypharmacy affected 42.1% of individuals aged 65 years and older (Kardas et al., 2021). Statins, antihypertensive drugs, atypical antipsychotics, and cholinesterase inhibitors have been shown to inhibit at least one common bacterial taxon of the gut microbiome (Totleben et al., 2025). Proton pump inhibitors increase gastric pH, leading to excessive growth of microorganisms such as *Streptococcus* and *Rothia* in the lower gastrointestinal tract, increased susceptibility to *Clostridioides difficile*, and reduced production of SCFAs. Conversely, biguanides like metformin increase the abundance of beneficial bacteria such as Akkermansia muciniphila, which stimulates mucin secretion. Antibiotics, particularly broad-spectrum agents, cause profound dysbiosis and increase the risk of opportunistic pathogen overgrowth. Corticosteroids reduce mucus production, while non-steroidal anti-inflammatory drugs (NSAIDs) reduce mucosal barrier integrity and increase susceptibility to injury by inhibiting cyclooxygenases. Furthermore, NSAID users exhibit an increased abundance of *Bacteroide*s and *Enterobacteriaceae*, alongside a decreased number of beneficial bacteria such as *Lactobacillus* and *Bifidobacterium* (Totleben et al., 2025).

#### 1.2.3 Classic UC-related mucosal abnormalities

In contrast to general aging, the classic pathophysiology of ulcerative colitis (UC) is characterized by a primary and profound breakdown of the mucosal barrier regardless of age. This dysbiosis is associated with a severe reduction in goblet cell numbers—the cells responsible for producing trefoil peptides, Fc-binding peptides, and mucins required for maintaining an intact and functional barrier (Kim and Ho, 2010). In patients with UC, reduced mucin content, altered mucin glycosylation, and decreased numbers and abnormal phenotypes of goblet cells are consistently demonstrated in inflamed intestinal regions. Specifically, goblet cells exhibit impaired mucin secretion in response to cholinergic agonists and prostaglandin E_2_ (Wong et al., 2025; van der Post et al., 2019). In long-standing UC, these chronic pro-inflammatory mechanisms and structural tissue remodeling persist over decades, reflecting the continuous interaction between chronic mucosal inflammation and the local microenvironment (Abraham and Cho, 2009).

#### 1.2.4 Distinct pathophysiological intersection in elderly-onset UC (EUC)

**FIGURE 1:**
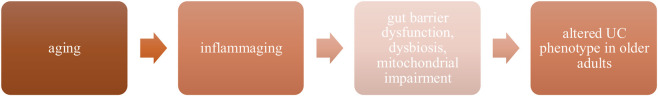
Schematic representation of the pathophysiological intersection in elderly-onset ulcerative colitis (EUC). Aging drives processes of immunosenescence and chronic low-grade systemic inflammation (“inflammaging”). These age-related changes, combined with polypharmacy and dietary shifts, lead to gut barrier dysfunction, significant dysbiosis (characterized by a decline in beneficial taxa and expansion of pathobionts), and epithelial mitochondrial impairment. The convergence of these mechanisms with classic mucosal abnormalities ultimately shapes the distinct and altered UC phenotype observed in older adults. Abbreviations: UC, ulcerative colitis; EUC, elderly-onset ulcerative colitis.

Crucially, elderly-onset UC (EUC) represents a distinct clinical and pathophysiological entity that arises at the unique intersection of the aforementioned processes. While young-onset UC is primarily driven by adaptive immune dysregulation, EUC is shaped by the convergence of core UC mechanisms with age-related physiological decline. A prominent example of this intersection is the age-related alteration in mitochondrial structure and function within the intestinal epithelium. In older individuals, increased expression of ALDH1L1 (Aldehyde Dehydrogenase 1 Family Member L1) modulates mitochondrial respiration (Zhang et al., 2025a). These age-related mitochondrial dysfunctions—characterized by abnormal morphology and the upregulation of inflammatory pathways (NLRP3/IL-1β inflammasome, TNF, and IL-17)—lead to an insufficient energy supply to colonocytes, resulting in impaired epithelial regeneration. Notably, these changes are highly pronounced and clinically definitive in elderly-onset UC, as evidenced by significantly increased expression of NLRP3 at both the mRNA and protein levels, enhanced caspase-1 activation, and elevated mature IL-1β detected via qPCR, Western blotting, and immunohistochemistry (Zhang et al., 2025a; Fu et al., 2024; Karłowicz et al., 2025). Ultimately, this age-aggravated mitochondrial dysfunction severely compromises the intestinal epithelial barrier by disrupting tight junctions, increasing permeability, and facilitating pathogen translocation that sustains both local and chronic systemic inflammation in EUC patients (Chernyavskij et al., 2023; Figure 1).

## 2 Diagnosis and clinical symptoms

### 2.1 Clinical manifestations—Colonic and extra-intestinal

In older patients, UC more frequently involves the left side of the colon, whereas in younger patients it is more commonly presented as isolated proctitis or pancolitis (Zhu and Ran, 2021; Nimmons and Limdi, 2016). The clinical manifestations in all age groups are mostly similar and include tenesmus, diarrhea, and hematochezia. Abdominal pain is more frequently observed in younger patients with UC, whereas anemia and weight loss are more common in elderly patients (Zammarchi et al., 2020; Zhu and Ran, 2021). Although the initial disease flare may have a more severe course, the symptoms in older adults are often less pronounced and may therefore be misinterpreted. Symptoms of comorbid conditions and the medications used may mask the manifestations of UC in older patients, leading to delayed diagnosis. For example, cardiovascular diseases, colonic ischemia, and the use of antiplatelet and anticoagulant medications, common in elderly patients, can further complicate the assessment of rectal bleeding. Complicated diverticular disease (including diverticulitis and diverticular bleeding), ischemic colitis, medication-associated diarrhea (related to NSAIDs and other agents), infectious diarrhea, radiation colopathy, and microscopic colitis should be considered in the differential diagnosis of ulcerative colitis in elderly patients. (Nimmons and Limdi, 2016; Kedia et al., 2018; Carbery et al., 2024). The spectrum of extraintestinal manifestations does not differ significantly in elderly patients, but they are less frequent compared to younger individuals. Retrospective studies have shown that 9.6% of patients aged 65 years or older experienced extraintestinal manifestations, compared with 19.2% of patients aged 40–60 years (Zammarchi et al., 2020; Zhu and Ran, 2021; Rogler et al., 2021). The most common UC extraintestinal manifestations include erythema nodosum, pyoderma gangrenosum, oral aphthous ulcers, arthritis (peripheral and axial), enthesitis, episcleritis and scleritis, anterior uveitis, and thromboembolism. Rare manifestations include autoimmune pancreatitis, autoimmune hepatitis, Sweet syndrome, orofacial granulomatosis, pneumonitis, and portal vein thrombosis (Nimmons and Limdi, 2016). Iritis and uveitis occur more frequently in IBD patients aged over 40 years compared to those under 40 years (Juillerat et al., 2020). It is also worth noting that extraintestinal manifestations of UC in older adults may be mistaken for other age-related conditions. Joint pain associated with UC is frequently underreported by patients or misinterpreted as age-related osteoarthritis, complicating the diagnostic and therapeutic approach. Therefore, a detailed understanding of the distinct musculoskeletal and rheumatological complexities in this population is highly warranted (Zammarchi et al., 2020).

#### 2.1.1 Musculoskeletal and rheumatological considerations

Extraintestinal manifestations involving the musculoskeletal system represent a major clinical challenge in geriatric UC management. Differentiating between IBD-associated arthropathy and age-related degenerative disorders is exceptionally difficult in patients aged ≥65 years. While osteoarthritis is nearly ubiquitous in this cohort, clinicians must actively screen for IBD-related peripheral arthritis (both Type 1, which parallels bowel activity, and Type 2, which runs an independent course) and axial spondyloarthritis (Harbord et al., 2016). A critical constraint in managing these overlapping rheumatological conditions in older adults is the strict “no-NSAID” policy. Non-steroidal anti-inflammatory drugs, while routinely used for general joint pain, are heavily restricted or contraindicated in elderly UC patients due to their well-documented risk of triggering acute intestinal flares, inducing mucosal ulceration, and significantly increasing the baseline risk of gastrointestinal bleeding, acute kidney injury, and major adverse cardiovascular events (MACE) (Nimmons and Limdi, 2016; Harbord et al., 2016). Consequently, when persistent musculoskeletal inflammation is present, the selection of advanced therapies must favor agents that provide concurrent control over both luminal and joint disease. Anti-TNF agents (such as infliximab or adalimumab) and anti-IL-12/23 inhibitors (such as ustekinumab) remain the preferred options for their established efficacy in systemic inflammatory arthropathies. In contrast, gut-selective therapies like vedolizumab are generally ineffective for rheumatological manifestations, which may lead to unresolved joint inflammation despite achieving clinical remission of colitis (Pabla et al., 2022). Ultimately, addressing these complex manifestations in older adults requires close multidisciplinary collaboration between gastroenterologists and rheumatologists to optimize targeted therapy while mitigating the risk of overtreatment and immunosuppressive complications.

### 2.2 Diagnosis

Diagnosing ulcerative colitis (UC) in elderly patients is significantly more challenging than in younger individuals due to an altered clinical presentation and a broader differential diagnosis. In clinical practice, initial symptoms in older adults are often non-specific or atypical; for instance, older patients may present with constipation or isolated rectal bleeding rather than classic severe diarrhea. This frequently leads to diagnostic delays, as symptoms can be mistakenly attributed to hemorrhoids, diverticular disease, or the use of anticoagulants. Furthermore, a wider range of age-associated conditions must be excluded during the diagnostic pathway. While colorectal cancer is a primary concern, clinicians must also differentiate elderly-onset UC from ischemic colitis, radiation proctitis, and medication-induced colitis, particularly secondary to the frequent use of non-steroidal anti-inflammatory drugs (NSAIDs) in this population. Unlike younger cohorts, where genetic mutations (such as variants within the HLA region, single-nucleotide polymorphisms, and CARD9 variants) play a more prominent role in susceptibility (Ha, 2012; Liang et al., 2024), environmental triggers appear dominant in late-onset disease. Notably, 60%–70% of older patients (aged 60 and above) with IBD diagnosed in older age have a history of tobacco use. In contrast to patients with elderly-onset IBD, smoking is not such a prominent characteristic in those diagnosed at a younger age (Ha, 2012). The standard diagnostic process relies on a complete colonoscopy with multiple mucosal biopsies for histopathological examination. However, completing this diagnostic pathway in older adults involves substantial clinical hurdles and risks. Beyond the procedural risks of endoscopy—such as a higher incidence of cardiopulmonary complications related to sedation and an increased risk of bowel perforation due to fragile mucosal walls—obtaining adequate bowel preparation remains a prerequisite for obtaining a reliable endoscopic assessment (Chinthala et al., 2025). Bowel preparation requires ingesting up to 4 L of a polyethylene glycol solution, which is often challenging for elderly patients, as they tend to consume low volumes of fluids daily. Spanish studies have shown that this issue affects up to 88% of individuals aged 60 years and older (Masot et al., 2020). Inadequate intake of the laxative solution results in insufficient bowel cleansing. Consequently, the reported adequacy of bowel preparation in patients aged ≥60 years ranges from 65.4% to 81.1%, compared with 77.3%–94.4% in younger cohorts (Chinthala et al., 2025). Additionally, in elderly patients, inadequate bowel preparation is influenced by factors such as weak anal sphincter tone, diabetes mellitus, chronic constipation, and cognitive or physical challenges in adhering to dietary and fluid recommendations. Crucially, the bowel preparation phase itself poses acute clinical risks in elderly patients, frequently triggering severe adverse events, including hypoglycemia, electrolyte disturbances, and profound orthostatic hypotension (Zhang et al., 2025b). Therefore, a tailored diagnostic approach with careful risk stratification and alternative preparation regimens is often required in geriatric clinical practice.

## 3 Complications and other special situations

### 3.1 *Clostridioides difficile* infection

Bloody diarrhea, particularly with sudden onset and associated fever, points to the differentiation between UC and infectious colitis. Pathogens such as *Shigella*, *Campylobacter*, *Salmonella*, *Escherichia coli*, and *Clostridioides difficile* may mimic the clinical presentation of UC and lead to misdiagnosis (Nimmons and Limdi, 2016). Older patients are at increased risk of *C. difficile* infection, particularly those residing in hospitals or long-term care facilities, and this risk is even more elevated in individuals with ulcerative colitis. Patients with IBD have up to a fivefold increased risk of *C*. *difficile* infection, and many of them are asymptomatic carriers (Gao et al., 2024; Dalal and Allegretti, 2021). An Irish study showed that the proportion of older patients who were asymptomatic carriers of *C*. *difficile* was 12% among those receiving short-term and long-term inpatient care, compared with 9.5% among older patients in ambulatory care and 1.6% among those recruited from the community (Rea et al., 2012). Patients with IBD are more susceptible to colonization by toxigenic taxa of *C. difficile* than patients without IBD (Vaughn et al., 2025). Antibiotic exposure may also concern IBD patients due to accompanying SIBO and gut dysbiosis (Vaughn et al., 2025). *Clostridioides difficile* colonization may remain asymptomatic or progress to active infection, depending on toxin production, interactions with intestinal receptors, and the immune system’s response to microbial products (Vaughn et al., 2025). *Clostridioides difficile* produces toxins that damage the intestinal epithelium, leading to profuse diarrhea, abdominal pain, and nausea. In individuals with UC, such an infection may lead to severe complications, including intestinal necrosis and systemic infection (Drobnik et al., 2024). Older patients with UC with *C. difficile* experience longer hospital stays, higher rates of intestinal resection, and increased mortality than patients aged <65 and without IBD (Dalal and Allegretti, 2021; Reznik et al., 2025). The AGA guidelines recommend treating the first mild episode of *C. difficile* in older UC adults with oral vancomycin or fidaxomicin, or metronidazole if the first two agents are unavailable. In contrast, severe or fulminant *C. difficile* infection requires intravenous metronidazole in combination with oral vancomycin. The ACG suggests considering extended vancomycin therapy for 21–41 days in patients with IBD, as this significantly reduces the risk of *C. difficile* infection recurrence. However, it should be noted that prolonged treatment with this antibiotic can disrupt the gut microbiome and consequently increase susceptibility to infection by other pathogens (Reznik et al., 2025).

### 3.2 Colorectal cancer risk in long-standing ulcerative colitis

The risk of colorectal cancer is directly proportional to the duration of the disease. Extensive and left-sided ulcerative colitis was associated with a higher risk of colorectal cancer compared to proctitis. Chronic inflammation impairs the regulatory mechanisms of colonic epithelial cells, thereby predisposing to dysplasia—a phenomenon known as ‘field cancerization.’ When the inflamed area involves extensive regions of the colon, the corresponding area of dysregulation is greater compared to the sporadic variant (Zhou et al., 2019). Age influences not only the risk but also the rate of colorectal cancer (CRC) development in UC. The median time to CRC was 10 years in patients diagnosed at ≥40 years, compared with 22 years in those diagnosed before age of 40 (Karvellas et al., 2007). Additionally, a large nationwide cohort analysis involving over 96,000 IBD patients demonstrated that individuals diagnosed with UC after the age of 70 had an approximately 15-fold higher incidence rate of CRC compared to the general population, representing the highest relative risk increase among the age sub-cohorts (Klepp et al., 2020). A meta-analysis further demonstrated that the cumulative risk of CRC was approximately 2%, 8%, and 18% after 10-, 20-, and 30 years following UC diagnosis, respectively (NICE, 2011). Current guidelines recommend initiating colonoscopic surveillance 8–10 years after IBD diagnosis. Nevertheless, earlier surveillance should be considered in patients diagnosed after the age of 40, and those diagnosed after the age of 70, in particular. Among older IBD patients, 29% developed colorectal cancer within 10 years of the disease diagnosis (NICE, 2011; Karvellas et al., 2007). After the surveillance starts, subsequent colonoscopy intervals must be strictly risk-stratified based on specific clinical features rather than age alone. High-risk patients—defined by the presence of concomitant primary sclerosing cholangitis (PSC), severe ongoing histological inflammation, a first-degree relative with CRC, or colonic strictures—require annual surveillance (every 1 year). Intermediate-risk individuals, including those with mild-to-moderate endoscopic inflammation or extensive post-inflammatory polyps, should undergo surveillance every 2–3 years, whereas low-risk patients with quiescent disease can safely extend the interval to every 5 years (Zhou et al., 2019; Gordon et al., 2023). Furthermore, in the geriatric population, the decision to continue surveillance must closely balance oncological benefits against procedural risks. Advanced age, frailty, and significant cardiorespiratory or renal comorbidities markedly increase the risk of colonoscopy-related adverse events, such as bowel perforation, severe dehydration from bowel preparation, and anesthesia-related complications. Therefore, international guidelines suggest that routine CRC surveillance should be individualized and may be safely discontinued in older adults when their overall life expectancy is estimated to be less than 5–10 years, ensuring that the burden of screening does not outweigh its preventative value (Gordon et al., 2023).

## 4 Therapeutic paths

In pharmacotherapy, clinicians should take into account potential drug–drug interactions, the severity of coexisting conditions, and other factors affecting patient safety, including cardiovascular risk as well as metabolic and renal comorbidities. A retrospective clinical study showed that approximately 34% of patients aged over 60 years with UC had cardiovascular diseases, while the prevalence of other comorbidities was approximately 18%. (Talar-Wojnarowska et al., 2024). The medications considered for the treatment of UC in older adults include 5-aminosalicylic acids (5-ASA), corticosteroids, immunosuppressive agents, biological therapies, and JAK inhibitors.

### 4.1 5-ASA

Proposed mechanisms of action of 5-ASA include free-radical scavenging and antioxidative effects. Anti-inflammatory activity has also been suggested through modulation of the cyclooxygenase and lipoxygenase pathways, as well as inhibitory effects on nuclear factor κB (NF-κB) and tumor necrosis factor (TNF) (Raine et al., 2021). These medications are used in the treatment of mild to moderate UC. It is considered first-line therapy and is the most widely used option in this population in as many as 70%–90% of older adults (Hong and Katz, 2021). 5-ASAs have a favorable safety profile; however, they may cause adverse effects such as nausea, abdominal pain, rash, nasopharyngitis, and arthralgia. Renal function should be monitored and regularly assessed during treatment - particular caution is required in patients who are taking one or more medications with nephrotoxic potential. Additionally, it should be noted that the prior history of liver disease may predispose patients treated with 5-ASA to hepatic dysfunction or failure. Therefore, the potential benefits and risks should be carefully evaluated before initiating therapy (Davis et al., 2024; Raine et al., 2021). PSC and UC frequently coexist—up to approximately 70%–80% of patients with PSC have IBD, mainly UC. The clinical registries have reported no published data, either positive or negative, regarding the direct impact of 5-ASA on PSC. 5-ASA acts locally in the gut, and its metabolites are rapidly inactivated in the liver, limiting potential effects on the bile ducts (Zhang et al., 2022; Kim et al., 2023). Proposed mechanisms of action of 5-ASA include free-radical scavenging and antioxidative effects. Anti-inflammatory activity has also been suggested through modulation of the cyclooxygenase and lipoxygenase pathways, as well as inhibitory effects on nuclear factor κB (NF-κB) and tumor necrosis factor (TNF) (Raine et al., 2021). These medications are used in the treatment of mild to moderate UC. It is considered first-line therapy and is the most widely used option in this population in as many as 70%–90% of older adults (Hong and Katz, 2021). 5-ASAs have a favorable safety profile; however, they may cause adverse effects such as nausea, abdominal pain, rash, nasopharyngitis, and arthralgia. Renal function should be monitored and regularly assessed during treatment - particular caution is required in patients who are taking one or more medications with nephrotoxic potential. Additionally, it should be noted that the prior history of liver disease may predispose patients treated with 5-ASA to hepatic dysfunction or failure. Therefore, the potential benefits and risks should be carefully evaluated before initiating therapy (Davis et al., 2024; Raine et al., 2021). PSC and UC frequently coexist—up to approximately 70%–80% of patients with PSC have IBD, mainly UC. The clinical registries have reported no published data, either positive or negative, regarding the direct impact of 5-ASA on PSC. 5-ASA acts locally in the gut, and its metabolites are rapidly inactivated in the liver, limiting potential effects on the bile ducts (Zhang et al., 2022; Kim et al., 2023). Concomitant medications frequently used in older adults can significantly alter 5-ASA pharmacokinetics and risk profiles. Proton pump inhibitors, antacids, and H2 receptor blockers alter gastric pH, which may lead to premature release of pH-dependent 5-ASA formulations in the upper gastrointestinal tract rather than the colon, potentially altering its therapeutic localization. 5-ASA increases the activity of heparin, thereby increasing the risk of bleeding, and also interacts with warfarin, leading to elevated INR and, consequently, an increased tendency to bleed. Crucially, 5-ASA acts as an inhibitor of thiopurine methyltransferase (TPMT), the enzyme responsible for thiopurine metabolism. Therefore, co-administration of 5-ASA with thiopurines (e.g., azathioprine, 6-mercaptopurine) does not reduce their efficacy, but instead significantly increases the accumulation of active thiopurine metabolites, markedly elevating the risk of severe, dose-dependent myelotoxicity and leukopenia. This interaction requires close hematological monitoring, particularly in the vulnerable elderly population (Nimmons and Limdi, 2016; Raine et al., 2021).

### 4.2 Corticosteroids

This drug class significantly increases the risk of serious infections. The use of corticosteroids in elderly patients with IBD is associated with a 2.3–2.8-fold increased risk of infections requiring hospitalization (Brassard et al., 2014). Consequently, the European Crohn’s and Colitis Organisation (ECCO) and international consensus guidelines strongly emphasize that systemic corticosteroids must be strictly reserved for short-term induction of remission, and their long-term use must be absolutely avoided in the older adult population. Steroids cause reduced bone density, thereby elevating the risk of fractures and osteoporosis, particularly in elderly patients. Long-term corticosteroid therapy can also lead to peptic ulcers and gastrointestinal bleeding—especially with concomitant use of NSAIDs. Steroids also impair wound healing, cause hypothalamic-pituitary-adrenal (HPA) axis suppression, steroid myopathy, cataract, and glaucoma, all of which further increase the clinical risk of hospitalization and complications in elderly patients (Raine et al., 2021; Narum et al., 2014). Additionally, corticosteroids contribute to diabetes development, fluid retention, hypertension, and psychiatric disturbances, primarily mood, delirium, and sleep disorders, the latter often detected in UC (Raine et al., 2021; Qazi and Farraye, 2019). When initiating corticosteroid treatment, a predefined therapeutic plan for the patient should be explained as an “exit strategy” (Sousa et al., 2023; Raine et al., 2021). Given the alarming cumulative toxicity profile in older adults, robust steroid-sparing strategies-such as the early introduction of gut-selective biologics (e.g., vedolizumab) or immunomodulators-must be prioritized immediately upon achieving clinical response to minimize exposure. Despite these considerable risks, studies indicate that within 10 years of UC diagnosis in the elderly, the probability of receiving corticosteroid therapy is 40%, compared to a 15% likelihood of receiving immunomodulatory or biological treatment (Mao et al., 2023). This difference, reflected by a higher frequency of corticosteroid use compared with biologic therapies, results from their long-standing clinical use, low cost, and wide availability, as well as from limited patient access to specialized IBD referral centers, for example, due to reduced mobility (Jeffrey and Noor, 2025; Rosiou et al., 2022). However, in modern geriatric gastroenterology, relying on prolonged steroid courses due to logistical or financial barriers is increasingly recognized as a major driver of preventable morbidity and hospitalization.

### 4.3 Anti‐TNF agents–Infliximab and adalimumab

Research studies demonstrate that younger patients with IBD or UC are more likely to receive biologic therapy compared with older adults. In a retrospective cohort of hospitalized UC patients, 36.5% of patients aged <65years received biologic therapy during admission compared with only 20.6% of those aged ≥65years. In the TARGET-IBD observational cohort, younger UC patients were also significantly more likely to use anti-TNF monotherapy than patients aged ≥65years (adjusted odds ratio 2.68) (Boyd et al., 2024; Barnes et al., 2022). This was related to concerns about potential risks and benefits of initiating biological therapy in the elderly, as possible complications, including infections and malignancies (Boyd et al., 2024; Cottone et al., 2011; Borren and Ananthakrishnan, 2019).

The biological therapies exert immunosuppressive effects and therefore increase the risk of infections and malignancies (Sousa et al., 2023). In adults over 65 years treated with biologic therapy, 11% of experienced severe infections, of whom 10% died, compared to 0.5% and 2% deaths among those not treated with biologics (Cottone et al., 2011). To minimize these severe infectious complications, strict pre-treatment screening protocols are mandatory in the elderly. Clinicians must screen for latent tuberculosis (via interferon-gamma release assays or tuberculin skin tests) and hepatitis B virus (HBV) serology before therapy initiation to prevent fatal reactivation. When considering anti-TNF agents, it is important to recognize that in patients over 60 years of age, the therapy is discontinued three times more frequently than in younger patients due to poor treatment tolerance, infections, or malignancies. Anti-TNF use is also accompanied by higher rates of hospitalization (mainly driven by infection-related complications) compared with those treated with vedolizumab or ustekinumab. Furthermore, specific age-related comorbidities impose absolute or relative contraindications; anti-TNF agents require extreme caution or are strictly contraindicated in older adults with moderate-to-severe congestive heart failure (NYHA class III/IV) due to risks of worsening cardiac function, as well as in patients with a history of demyelinating diseases such as multiple sclerosis. However, despite these systemic risks, anti-TNF monoclonal antibodies provide distinct clinical advantages in specific phenotypes, as they are highly beneficial in managing coexisting extraintestinal manifestations frequently seen in older adults, including uveitis, peripheral arthritis, and axial spondyloarthritis-like disease.

### 4.4 Gut-selective and targeted cytokine biologics–Vedolizumab and ustekinumab

In contrast to systemic anti-TNF agents, newer advanced therapies offer more targeted mechanisms of action, which is highly advantageous in the older adult population. Vedolizumab is a humanized monoclonal antibody that binds exclusively to the integrin, blocking the infiltration of inflammatory lymphocytes into the gastrointestinal mucosa. Due to this strictly gut-selective mechanism, vedolizumab avoids systemic immunosuppression, resulting in an exceptionally favorable safety profile with a significantly lower risk of serious infections and malignancies in elderly patients. This is reflected in clinical practice data; for instance, in a single-institution retrospective cohort of IBD patients aged over 60 years, vedolizumab was discontinued significantly less often than anti-TNF therapy, with 51.9% of patients remaining on treatment after 1 year compared to just 25.9% receiving TNF antagonists (Pabla et al., 2022). However, a major clinical limitation of its gut-selective nature is its reduced efficacy against systemic extraintestinal manifestations (EIMs); vedolizumab is generally ineffective for coexisting IBD-related conditions such as uveitis, peripheral arthritis, or axial spondyloarthritis.

Ustekinumab, a monoclonal antibody targeting the shared p40 subunit of interleukins 12 and 23 (IL-12/23), provides a systemic alternative with a targeted pathway. While it exerts systemic anti-inflammatory actions, large registry studies and real-world geriatric data have demonstrated that ustekinumab possesses a remarkably safe profile in older adults, carrying a significantly lower risk of severe infections and hospitalization compared to anti-TNF agents. Unlike vedolizumab, ustekinumab’s systemic mechanism allows it to be highly effective in managing both intestinal inflammation and concurrent extraintestinal manifestations, particularly peripheral arthritis and psoriasis-like skin lesions. Therefore, while vedolizumab represents the ideal choice for older adults with isolated, moderate-to-severe bowel disease, ustekinumab serves as a preferred, safe systemic option when extraintestinal manifestations require concomitant treatment.

### 4.5 Thiopurines–azathioprine and 6-mercaptopurine (6-MP)

Thiopurines are prodrugs of purine analogs of hypoxanthine and have been used to maintain remission in moderate to severe UC in older adults, being prescribed in 5%–26% of elderly UC patients (Zhu and Ran, 2021). They are most commonly administered long-term, i.e., for more than 12 months, and it was indicated that they reduce the risk of colectomy by approximately 70%. However, using thiopurines in the elderly requires strict risk stratification and pretreatment screening, which are critical in geriatric clinical practice. Before initiating therapy, pharmacogenetic testing for Thiopurine Methyltransferase (TPMT) and Nucleoside Diphosphate-Linked Moiety X-Type Motif 15 (NUDT15) polymorphisms should be performed to identify patients at a high risk of developing severe, life-threatening bone marrow suppression. Furthermore, evaluating baseline Epstein-Barr Virus (EBV) serological status is essential, as EBV-seronegative older adults face a significantly elevated risk of developing fatal post-mononucleosis lymphoproliferative disorders or lymphomas under thiopurine immunosuppression. Even in EBV-seropositive individuals, they are connected to the high risk of infections, non-melanoma skin cancers (NMSC), myeloproliferative disorders, and hepatosplenic T-cell lymphoma (HSTCL). Consequently, active dermatological screening for NMSC is recommended annually. The most common adverse effects of thiopurines in patients over 60 years of age are myelotoxicity, gastrointestinal intolerance, and hepatotoxicity. To mitigate these severe risks, continuous clinical vigilance is required, including regular complete blood count (CBC) monitoring to detect delayed myelosuppression and frequent liver function monitoring. Prolonged use beyond 6 months increases the incidence of nodular regenerative hyperplasia (NRH) of the liver and symptomatic portal hypertension, including ascites and varices. Contraindications to thiopurine therapy include renal disease or reduced renal clearance, a history of malignancy or lymphoma, and concomitant use of xanthine oxidase inhibitors (Zhu and Ran, 2021; Raine et al., 2021). According to ECCO guidelines, thiopurines are generally avoided as first-line therapy in older patients (>65 years) due to safety concerns associated with infection and malignancy risks in the elderly (Raine et al., 2021). In one analysis, overall adverse events occurred in 43.4% of patients who initiated thiopurines at ≥60 years compared with 29.7% in younger patients, with higher rates of infections (3.6% vs. 2.0%) and malignancies (1.5% vs. 0.2%) in the older group. Furthermore, pooled data from multiple studies show an almost five-fold increased risk of lymphoma in IBD patients treated with thiopurines (Strigáč et al., 2024; Kotlyar et al., 2015). Thiopurine metabolites contribute as well to potential adverse effects and drug interactions with warfarin, mesalazine, furosemide, and allopurinol—medications often taken by individuals over 65 years of age (Raine et al., 2021). A 2012 analysis of the adverse effect profile of 6-mercaptopurine/azathioprine in elderly IBD patients, including 41 patients with a mean age of 70.4 years, showed that 73.2% experienced at least one adverse event, while 19.5% experienced multiple adverse events (Strigáč et al., 2024; Clement et al., 2023). The most common adverse events, in order of frequency, were: leukopenia/neutropenia (16 patients, 39.0%), elevated liver enzymes/hepatotoxicity (10 patients, 24.4%), gastrointestinal intolerance (nausea, vomiting, abdominal pain; 8 patients, 19.5%), rash or hypersensitivity reactions (5 patients, 12.2%), infections (5 patients, 12.2%), and pancytopenia or severe cytopenia (4 patients, 9.8%). Additionally, malignancies (including pancreatic cancer, acute myeloid leukemia/myelodysplastic syndrome, and kidney/bladder cancers) were observed in 2 patients (4.9%), and 4 patients (9.8%) discontinued therapy due to severe adverse events. Therefore, the ECCO recommendation is to avoid thiopurines as first-line therapy in patients over 65 years of age (Strigáč et al., 2024).

### 4.6 JAK inhibitor–tofacitinib, upadacitinib, and filgotinib

Tofacitinib inhibits intracellular Janus kinase (JAK) tyrosine kinases, which ar JAK inhibitors are orally available small molecules that have revolutionized the management of moderate-to-severe UC due to their rapid onset of action. While early clinical experience was dominated by tofacitinib (a pan-JAK inhibitor), modern therapeutic strategies increasingly incorporate highly selective JAK1 inhibitors, such as upadacitinib and filgotinib. However, because these agents alter cytokine signaling, their use in older adults demands stringent clinical evaluation and rigorous pre-treatment risk stratification. JAK inhibitors significantly increase the risk of serious and opportunistic infections in geriatric patients; the most commonly reported involve the upper respiratory and urinary tracts, alongside frequent reactivations of CMV, EBV, BKV, and latent tuberculosis. Notably, a dose-dependent, significantly elevated risk of Herpes Zoster (shingles) infection is a class-wide concern that is particularly pronounced in elderly cohorts, necessitating proactive vaccination strategies.

The safety profile of JAK inhibitors has been heavily scrutinized following the landmarks of the ORAL Surveillance safety trial. Although this study primarily evaluated tofacitinib in patients with rheumatoid arthritis aged 50 years or older with at least one cardiovascular risk factor, major regulatory agencies (such as the EMA/PRAC and FDA) have extended its black-box warnings to all JAK inhibitors across all chronic inflammatory indications, including ulcerative colitis (European Medicines Agency, 2022; Sands et al., 2020). Consequently, in modern clinical practice, JAK inhibitors must only be initiated in older adults if no suitable therapeutic alternatives are available. Prior to prescription, clinicians must execute a mandatory risk stratification protocol focusing on five key axes: advanced age (particularly patients aged ≥65 years), a current or prolonged past history of smoking, established cardiovascular risk factors (including a history of atherosclerotic cardiovascular disease, myocardial infarction, or stroke), a high baseline risk for venous thromboembolism (VTE), and any prior history of malignancy (European Medicines Agency, 2022; Ytterberg et al., 2022).

The pro-thrombotic mechanism of tofacitinib and the wider JAK inhibitor class is not fully understood, but current hypotheses suggest that while the drug reduces inflammation by inhibiting cytokine signaling (e.g., IL-6, IFNγ), it does not prevent the pro-thrombotic effects of cytokines on endothelial cells, including the upregulation of adhesion molecules and coagulation factors. This may partially explain the observed increased risk of thrombosis in patients with active inflammation treated with JAK inhibitors (Zavoriti et al., 2025). Furthermore, JAK inhibition frequently causes hyperlipidemia, characterized by dose-dependent increases in total cholesterol, LDL-C, and HDL-C levels, which is particularly concerning in patients with elevated cardiovascular risk (European Medicines Agency, 2022; Ytterberg et al., 2022). Clinical studies have demonstrated a clear increase in the incidence of pulmonary embolism and related mortality with JAK inhibitors, especially at higher induction doses (Raine et al., 2021).

The venous thromboembolism (VTE) occurred in approximately 5%–6% of patients aged ≥65 years treated with tofacitinib, compared with around 4% of younger patients. In high-risk settings, such as surgery or immobilization, the incidence of thromboembolic events is significantly higher. Among older patients undergoing colectomy or proctocolectomy who had previously received tofacitinib, VTE occurred in 22% of patients, compared with 8.5% in the control group (Russell et al., 2024). Importantly, recent real-world data and sub-analyses regarding upadacitinib use in IBD suggest favorable real-world efficacy and relatively acceptable short-to-medium-term safety in older adults, showing no disproportionate increases in MACE, VTE, or malignancies compared to younger patients when strict baseline risk exclusion is applied. Nevertheless, given that IBD treatment requires higher maintenance doses than other inflammatory diseases, meticulous cardiovascular and oncological screening remains an ongoing mandate for all elderly individuals on any JAK inhibitor therapy.

### 4.7 IL-23 inhibitors

IL-23 inhibitors such as mirikizumab and risankizumab represent promising biologic options for moderate-to-severe ulcerative colitis, with systematic reviews and real-world evidence demonstrating significant clinical remission and favorable safety profiles. Although specific data in elderly patients are limited, available evidence suggests acceptable tolerability and effectiveness, positioning these agents as valuable therapeutic alternatives, particularly for individuals at higher risk of infection or adverse events associated with other biologics (Ela et al., 2025; Takagi et al., 2025).

### 4.8 S1P receptor modulators–Ozanimod and etrasimod

Sphingosine 1-phosphate (S1P) receptor modulators are orally administered small molecules that selectively inhibit intracellular trafficking by trapping lymphocytes within peripheral lymph nodes, thereby preventing their migration into the inflamed gastrointestinal mucosa. While these agents provide a convenient, non-immunogenic alternative to biological therapies, their use in older adults must be carefully evaluated due to distinct systemic adverse effects. Geriatric patients exhibit a heightened vulnerability to S1P-induced cardiovascular complications, particularly bradycardia and atrioventricular conduction blocks. Consequently, a baseline electrocardiogram (ECG) is a mandatory component of the pre-treatment assessment for all older adults, and these medications are strictly contraindicated in individuals with a history of recent myocardial infarction, unstable angina, stroke, transient ischemic attack (TIA), or advanced decompensated heart failure within the preceding 6 months. Furthermore, S1P modulator therapy is associated with a dose-dependent risk of macular edema, which is significantly amplified in older adults, especially those with pre-existing diabetes mellitus or a history of uveitis. Clinical protocols require comprehensive baseline ophthalmic examinations followed by periodic regular monitoring during treatment. Due to the class-wide risk of hepatotoxicity and opportunistic infections, pre-treatment screening must also include liver function tests to identify liver enzyme abnormalities and serological screening for active varicella-zoster virus (VZV) immunity. Although real-world data suggest that S1P modulators carry a lower overall infection risk compared with anti-TNF agents, their severe cardiovascular and ophthalmic restrictions necessitate meticulous patient selection. Therefore, similar to other small molecules, the general clinical consensus is to avoid S1P receptor modulators in elderly patients with significant cardiac, metabolic, or ophthalmologic comorbidities, reserving them primarily for ‘fit’ older individuals.

### 4.9 Clinical summary and management algorithm

To bridge the gap between individual drug classes and clinical decision-making, choice of therapy in older adults must be strictly guided by both disease severity and the patient’s overall health status (distinguishing between ‘fit’ and ‘frail’ elderly individuals). Table 1 provides a practical, clinical summary of this treatment algorithm.

**TABLE 1:** Risk-stratified management algorithm for older adults with ulcerative colitis (Lamb et al., 2019; Lobatón et al., 2015).

Disease severity	‘Fit’ older Adults (low comorbidities, independent)	‘Frail’ older Adults (multiple comorbidities, polypharmacy, vulnerable)
Mild to moderate	• First-line: Topical or oral 5-ASA. • Second-line: Budesonide MMX or short-course systemic corticosteroids	• First-line: 5-ASA (monitor adherence if cognitive decline is present) • Second-line: Prefer local budesonide. Systemic corticosteroids require extreme caution (high risk of hyperglycemia, delirium, and fractures; mandatory bone protection)
Moderate to severe	• Advanced therapies: Anti-TNF agents (infliximab, adalimumab), anti-integrins (vedolizumab), anti-IL-12/23 (ustekinumab, risankizumab), or S1P receptor modulators (ozanimod, etrasimod) • JAK inhibitors (tofacitinib, upadacitinib, filgotinib) require strict screening for cardiovascular, smoking, and thromboembolic risks	• Preferred advanced therapies: Gut-selective agents (vedolizumab) or targeted biologics with favorable safety (ustekinumab) due to significantly lower systemic infection and malignancy risks • Avoid anti-TNF agents, thiopurines, and JAK inhibitors if possible due to high systemic toxicity, infection, or cardiovascular risks
Severe/Refractory	• Standard rescue therapy or timely surgical consultation for colectomy	• Early surgical consultation for colectomy is critical. Delaying surgery with multiple consecutive medical trials significantly increases mortality

## 5 Surgery in elderly UC patients

The indications for surgical intervention in older patients with UC are very similar to those in younger individuals. They include failed pharmacotherapy, detected neoplasia or colorectal cancer, bowel perforation, and toxic megacolon (Kampka et al., 2023; Stephens et al., 2025). Population-based studies have shown a sustained decline in colectomy rates among patients with UC over recent decades, largely due to advances in medical management, particularly the introduction of biologic therapies. Although there are no published studies specifically reporting a temporal decline in colectomy rates exclusively among elderly-onset ulcerative colitis patients, population-based research indicates an overall decrease in colectomy rates for UC in the biologic era. In addition, older patients are often poor surgical candidates due to concomitant diseases (Parragi et al., 2018; Giddings et al., 2023).

In a retrospective cohort clinical study, among patients undergoing colectomy, emergency procedures were significantly more frequent in older adults, with 50% of surgeries (7 of 14) classified as urgent, compared with only 12.8% (5 of 39) in younger adults. This indicates that older patients with UC are more likely to require urgent surgical intervention than their younger counterparts. The higher proportion of urgent surgical interventions among older patients may be associated with delayed or less intensive medical treatment in this age group compared to younger patients. The older patients more frequently develop complications such as bowel perforation, toxic megacolon, or abscess formation, which necessitate urgent surgical intervention (Boyd et al., 2024). In addition, available cohort studies suggest that older adults are much less likely to receive restorative procedures, such as an ileal pouch–anal anastomosis, following colectomy. In one study of patients hospitalized after J-pouch for severe UC, none of the older patients (≥65 years) received digestive tract reconstruction compared to 77% reconstruction in younger patients (Boyd et al., 2024). In older adults, beyond comorbidities, unfavorable prognostic factors include poor nutritional status, drug–drug interactions, and limited mobility. Frequent postoperative complications in this age group include wound dehiscence, shock, pneumonia, prolonged ventilator dependence, and urinary tract infections. An increased risk of myocardial infarction, sudden cardiac arrest, and greater perioperative transfusion requirements should also be considered (Bentrem et al., 2009; Alselaim et al., 2023). In elderly UC patients, colectomy is performed less frequently despite similar indications as in younger adults. This is largely due to comorbidities, polypharmacy, poor nutritional status, and limited mobility, which increase surgical risk and complicate perioperative management. Consequently, older patients are more often managed conservatively, which frequently leads to delayed interventions and a higher proportion of high-risk emergency surgeries rather than planned, elective procedures (Boyd et al., 2024). However, it is critical to emphasize that chronological age alone should not lead to excessive delays when surgical intervention is clinically indicated. Cycling through multiple consecutive medical therapies in a refractory geriatric patient often results in deep immunosuppression and physical deconditioning, which worsens outcomes. A balanced clinical approach mandates early, multidisciplinary surgical consultation; timely, elective colectomy should be pursued proactively in ‘fit’ older individuals and viewed as a definitive therapeutic strategy to optimize survival rather than a failure of medical management (Ananthakrishnan et al., 2020).

## 6 Nutrition, rehabilitation, and other supportive care aspects

### 6.1 Nutrition

Older adults are at increased risk of malnutrition, and this risk is further amplified in ulcerative colitis. In contrast, malnutrition in younger patients with UC is more often directly related to disease activity, restrictive diets, or increased metabolic demands during flares, rather than to age-related physiological and functional factors (Schreiner et al., 2020). Multimorbidity, polypharmacy, poor dentition, and neuropsychological factors (such as depression and anxiety) all contribute to this pathology. With advancing age, gastric capacity decreases, and gastric emptying becomes delayed; taste bud function may also be impaired. There is also evidence indicating that age-related changes in gut hormones, including increased cholecystokinin and reduced ghrelin levels, may contribute to early satiety during meals in older adults, potentially affecting nutritional intake (Nifli, 2018; Eder et al., 2019). Socioeconomic factors also play a significant role, including loneliness, poverty, social isolation, and limited ability to shop independently due to reduced physical capacity and architectural barriers. According to the Roubenoff classification, the causes of weight loss are categorized based on their underlying mechanisms, which include cachexia, wasting, and sarcopenia (Eder et al., 2019). Due to the diverse mechanisms underlying malnutrition, the use of indicators that do not account for body composition (such as BMI), the lack of assessment of micro- and macronutrient levels, and the limited consideration of recent disease activity, considerable variability exists in estimates of the prevalence of malnutrition among patients with UC, 18%–62% (Eder et al., 2019). Malnourished patients and obese individuals with hidden fat-free mass deficiency are more likely to experience frequent hospitalizations, including those related to disease exacerbations. Because catabolism is intensified during active inflammation–in active UC, in particular-protein intake should be increased from 1 g/kg body weight per day (the recommended intake for healthy individuals and for patients in remission) to 1.2–1.5 g/kg body weight per day. Caloric intake should be 30–35 kcal/kg/day, similar to that recommended for healthy individuals. Additionally, in the acute phase, patients often reduce their food intake because of pain and discomfort. Due to malabsorption, diarrhea, and reduced dietary intake, micronutrient levels should be assessed at least once a year. It is important to note that in active disease, acute inflammation alters laboratory values - serum ferritin and copper concentrations increase, whereas selenium, folate, and zinc levels decrease. Therefore, reliable and accurate conclusions regarding micronutrient deficiencies can be drawn only when these assessments are performed during disease remission. A particular situation is iron deficiency, for which specific diagnostic criteria differ depending on the degree of inflammatory activity-frequency of measurements should be adjusted according to disease activity: in remission or mild disease, every 6–12 months, and in active disease, at least every 3 months. It is generally accepted that in patients without clinical, endoscopic, or biochemical evidence of active disease, a serum ferritin level below 30 μg/L meets the criterion for iron deficiency. In the presence of inflammation, however, a serum ferritin level of up to 100 μg/L may still indicate iron deficiency. In cases of remission or mild disease, complete blood count and fecal calprotectin (FC) should be assessed regularly every few months. In the presence of new or worsening symptoms, FC, CRP, and stool tests should be promptly performed to exclude infection (Kucharzik et al., 2025; Godala et al., 2025).

There is no strong scientific evidence that the specific diet prolongs remission. There are some indications of benefits from the Mediterranean or DASH diets, but these findings are more supported by their favorable metabolic effects in older adults rather than a direct impact on UC clinical course (Bischoff et al., 2023; Eder et al., 2019; Dignass et al., 2015).

### 6.2 Rehabilitation and osteoporosis

Patients with UC frequently reduce their physical activity, which is attributable to the belief that exercise may exacerbate the disease and increase the general malaise or discomfort. Actually, physical activity enables patients to improve their quality of life, prolong periods of remission, reduce both colonic and extraintestinal symptoms, and counteract muscle atrophy. Exercise programs should be individually tailored in collaboration with a physician and a physiotherapist, with careful consideration of the patient’s clinical condition, functional abilities, and overall tolerance to physical activity (Kovács et al., 2025; Gatt et al., 2019).

In the population-based study from Denmark, it was shown that among older patients with UC, over one-third of women had osteoporosis, while in men, it was present in approximately 13% (Attauabi et al., 2025). Many patients have vitamin D deficiencies, which, in addition to regulating calcium and phosphorus levels (thereby affecting bone density), also exert beneficial immunomodulatory effects. Patients treated with corticosteroids for 3 months or longer should undergo osteoporosis screening using dual-energy X-ray absorptiometry (DXA). Vitamin D_3_ supplementation is recommended, along with glucocorticoid-sparing strategies. In cases of established osteoporosis, treatment options may include bisphosphonates, denosumab, parathyroid hormone analogs, or selective estrogen receptor modulators (Kreienbuehl et al., 2024; Vernia et al., 2025; Ali et al., 2009; Yin et al., 2022). Individuals at risk due to long-term corticosteroid use and patients with osteopenia are recommended to receive calcium at a dose of 500–1000 mg/day and vitamin D at a dose of 800–1000 IU/day. According to Polish and geriatric recommendations, older adults require higher vitamin D supplementation due to age-related factors: adults aged 65–75 years should receive 1000–2000 IU/day, while those over 75 years should receive 2000–4000 IU/day, reflecting reduced cutaneous synthesis and increased risk of osteoporosis. Specific guidance on vitamin D dosing in older patients with ulcerative colitis is not established (Marcin, 2025; Eder et al., 2023).

### 6.3 Vaccination

In IBD, there is increased susceptibility to infectious diseases. Therefore, older UC patients, particularly those receiving immunomodulators or advanced therapies, require a systematic baseline infection-risk assessment prior to treatment initiation. Due to immunosenescence and potential comorbidities, geriatric individuals exhibit a significantly higher risk for opportunistic infections, reactivation of latent pathogens (such as tuberculosis or HBV), and severe viral complications. Some of these infections can be prevented through appropriate vaccinations. Vaccination recommendations for older patients with IBD are similar to those for younger patients with IBD, but they demand stricter clinical timing, ideally being completed 2–4 weeks before starting advanced therapy. ECCO guidelines recommend annual influenza vaccination for all patients with IBD, and SARS-CoV-2 vaccination is included as part of the vaccination strategy for IBD patients according to national guidelines (Kucharzik et al., 2021; Magro et al., 2020). Furthermore, older adults must receive a pneumococcal vaccination series (utilizing sequential PCV15/20 and PPSV23) and respiratory syncytial virus (RSV) immunizations to reduce severe respiratory hospitalizations. Given the exceptionally high, dose-dependent risk of shingles associated with both advanced age and JAK inhibitor therapy, the non-live recombinant zoster vaccine (Shingrix) is highly mandated. Crucially, clinicians must remember that live vaccines (e.g., yellow fever, MMR) are strictly contraindicated once any immunosuppressive or biological therapy has commenced (Raine et al., 2021; Singh et al., 2023).

### 6.4 Mental health aspects

Risk factors for depression in the general elderly population include older age, a history of depression, the death of a spouse, health-related factors, and comorbid anxiety. Individuals with IBD represent one of the chronic disease groups at increased risk of depression. In an online cohort study conducted by the Crohn’s and Colitis Foundation of America Partners, using the Geriatric Depression Scale (GDS), the prevalence of depression in IBD was 22.6%, although these data were obtained from a broader IBD cohort including Crohn’s disease, they provide a valuable clinical extrapolation for older UC patients. (Long et al., 2014). Mental health influences the course of UC, and patients with cognitive deficits and depression exhibited more severe disease symptoms compared to those without such impairments. Additionally, medical recommendations were followed more consistently by patients without psychiatric or cognitive deficits. Depression was also associated with poorer treatment outcomes and an increased risk of relapses or hospitalizations (Hu et al., 2021).

## 7 Summary

The rising prevalence of elderly-onset ulcerative colitis presents unique diagnostic and therapeutic challenges that demand a paradigm shift toward geriatric-focused IBD care. Clinicians must maintain a high index of suspicion, as nonspecific presentations often mimic other age-related conditions, and diagnostic delays are further compounded by practical difficulties like inadequate bowel preparation. Managing UC in this vulnerable population requires moving away from standardized algorithms in favor of a personalized, multidisciplinary approach that accounts for extensive multimorbidity, polypharmacy, and altered drug pharmacokinetics.

Safe therapeutic navigation involves strictly limiting corticosteroid exposure, avoiding thiopurines, and carefully weighing the risk-benefit profiles of advanced therapies, where gut-selective biologics like vedolizumab often offer a safer alternative to anti-TNF agents. Furthermore, the disproportionately high risk of colorectal cancer in patients diagnosed later in life justifies aggressive, early surveillance strategies, alongside comprehensive supportive care addressing nutritional deficits, bone health, and mental health.

To optimize outcomes in geriatric IBD, future clinical research must focus on establishing age-specific treatment guidelines and developing reliable frailty indices rather than relying solely on chronological age. There is an urgent need for clinical trials that actively include older adults with comorbidities to better evaluate the safety of novel small molecules and biologics. Additionally, exploring the intersection of immunosenescence, age-related gut microbiome dysbiosis, and targeted non-immunosuppressive therapies represents a critical Frontier in establishing safer, long-term remission strategies for older patients.
